# Thanksgiving and Christmas gatherings before the 2020–21 winter surge of COVID-19 in the United States

**DOI:** 10.1016/j.pmedr.2022.101911

**Published:** 2022-07-21

**Authors:** Tim A. Bruckner, Abhery Das, Greg J. Duncan

**Affiliations:** a Program in Public Health, University of California, Irvine, United States; b Center for Population, Inequality, and Policy, University of California, Irvine, United States; c School of Education, University of California, Irvine, United States

**Keywords:** COVID-19, Holiday gatherings, Pandemic, Coronavirus, Risky holiday behavior

## Abstract

**Objective:**

COVID-19 in the US disproportionately affected, and continues to affect, racial/ethnic minorities. Although risky social gatherings for Thanksgiving and Christmas in 2020 contributed substantially to the “winter surge” in cases and deaths, no research examines potential racial/ethnic differences in behaviors related to holiday gatherings.

**Design:**

We used the Understanding America Survey (UAS) - Coronavirus Tracking, a nationally representative study of US adults, to examine associations between race/ethnicity and risky holiday gathering behavior (i.e., gathering with non-household members and with little to no social distancing or mask-wearing). We applied logistic regression models to examine racial/ethnic and socioeconomic differences in risky holiday gatherings while accounting for a person’s pre-holiday perception of COVID-19 risk as well as related behaviors.

**Results:**

Non-Hispanic Black adults showed a lower prevalence of attending a risky Thanksgiving gathering than did non-Hispanic White adults (15 % vs 43 %, p <.001). The magnitude of this racial/ethnic difference was also found for risky Christmas gatherings. Hispanic and “Other” race/ethnicity adults also appeared less likely than non-Hispanic whites to attend a risky holiday gathering. Higher-income households attended a risky holiday gathering more frequently, when compared with lower income households (p <.001). Logistic regression results, which controlled for other COVID-19 related behaviors, support these main findings.

**Conclusions:**

Racial/ethnic minorities, and non-Hispanic Black adults in particular, appeared least likely to have engaged in risky holiday gatherings in late 2020. If replicated, our findings appear consistent with the notion that behavioral modification among racial/ethnic minorities may have reduced the intensity of the 2020/21 “winter surge” in COVID-19.

## 1 Introduction

Case rates of COVID-19 in the US peaked in mid-January of 2021 and 2022 (Data Tracker, 2020). These peaks occurred approximately-two weeks after Christmas and Thanksgiving, respectively. Before the late 2020 holidays, media reports speculated that travel and large gatherings for these events may create a “winter surge” of COVID-19 above and beyond the seasonally expected increase. The Centers for Disease Control and Prevention (CDC), moreover, issued advisories against travel and holiday gatherings. Despite these advisories, airline and vehicular travel increased substantially around these holidays (Transportation Security Administration, 2021, Fernandez et al., 2020, Groves, 2021).

Before – and after – the 2020 holidays, COVID-19 in the US disproportionately affected racial/ethnic minorities and persons of lower socioeconomic status (SES). (Lopez et al., 2021, CDC, 2020) These disparities reportedly arise in part from elevated COVID-19 exposure for those working in “frontline” jobs and those living in dense housing and neighborhoods. Recognition of COVID-19 as a disease of disparity has led the CDC to issue a call regarding redressing health inequities along SES and racial/ethnic lines (Lopez et al., 2021, CDC, 2020).

Researchers who model COVID-19 transmission across place and time argue that human mobility patterns and prevalence of engagement in risky behavior better predicts COVID-19 infection than does knowledge of the spatial distribution of vulnerable populations (Carroll and Prentice, 2021). As it relates to holiday gatherings and the winter surge, such a perspective would call for a careful analysis of groups that engaged in holiday gatherings. Surprisingly, we know of no nationally representative study that evaluates racial/ethnic and SES differences in behaviors related to holiday gatherings. Two subnational surveys find that younger aged persons (<35 years) engaged in relatively more holiday gatherings with non-family members (Peacock et al., 2020). But these surveys under-represented low-SES persons as well as racial/ethnic minorities and therefore could not address holiday behaviors across these important groups (Peacock et al., 2020).

We build on this previous work in two important ways. First, we use a nationally representative study of US adults to examine the associations between SES and race/ethnicity and 2020 holiday gathering behavior. The literature does not provide a clear directional hypothesis in this area. The public health perspective on human behavior posits that a person’s assessment of risk affects their decisions about protective behaviors (Rosenstock, 1974). From this perspective, low SES and historically disadvantaged racial/ethnic groups may engage less in risky holiday gatherings if they know of close contacts who died of COVID-19 and therefore fear infection. By contrast, non-Hispanic Black adults report larger fictive kin networks and broader co-residence networks with extended relatives than do non-Hispanic whites (Taylor et al., 2013, Cross, 2018). In addition, Hispanic families report more extensive multigenerational households and local family networks than do whites (Cross, 2018, Cohen and Casper, 2002). These circumstances may have led to a greater social pressure (and desire) for Black and Hispanic adults, as compared with white adults, to gather for the holidays.

Second, our work examines holiday gatherings as a behavior that is potentially distinct from other COVID-19 related behaviors. The decision to gather with extended family and friends for the holidays may differ fundamentally from decisions to, for instance, get vaccinated, wear a mask, or maintain a 6-ft distance from others. The Understanding America Study (UAS) contains information gathered across 2020 on COVID-19 risk perceptions and related behaviors, which allows us to examine SES and racial/ethnic predictors of 2020 holiday gatherings while accounting for a person’s pre-holiday perception of COVID-19 risk as well as related risk behaviors.

## 2 Materials and methods

### 2.1 Data

We drew our study sample from participants in the Understanding America Survey (UAS) Coronavirus Tracking Survey, a probability-based internet panel of adults in the US. Details regarding sampling methodology and survey design appear in the Appendix and the UAS website (https://uasdata.usc.edu). The longitudinal nature of the UAS, combined with its representative nature and timely data releases, has led to its widespread use in peer-reviewed publications (Understanding America Study, 2021). Response rates for the UAS range from 67.1 % to 80.4 %, based on the survey wave (Understanding America Study, 2021).

We used data from multiple waves of the study. We used waves 1–16 (March 10, 2020–November 11, 2020) to capture pre-holiday data on COVID-19 perceptions and behaviors prior to the holidays and Wave 27 (April 14, 2021–March 25, 2021) for retrospectively reported information on holiday risky behaviors. Our analytic sample comprised 5,906 participants who provided complete data on the variables of interest. Respondents participated in greater than 97 % of questions on sociodemographic characteristics and holiday risk behavior (Understanding America Study, 2021).

### 2.2 Variables

Our key dependent variable is drawn from retrospective reports from Wave 27 of behaviors related to holiday gatherings of Thanksgiving and Christmas in late 2020. The key Thanksgiving question read: “Thinking back to this past Thanksgiving, did you get together with friends or relatives who do not live with you to celebrate?” Participants could respond “yes” or “no.” “Yes” responses led to this follow-up question: “When you were inside, how many of you wore masks and socially distanced?” Response choices included “All of us,” “Most of us,” “Some of us,” and “None of us.” UAS included these same questions for Christmas gathering behaviors, with the same wording as above save for using “Christmas” instead of “Thanksgiving.”.

We created a binary indicator for risky Thanksgiving behavior in which we assigned a “0” to respondents if they 1) did not attend Thanksgiving; or 2) attended Thanksgiving and all or most guests wore masks and socially distanced while indoors. We then assigned a “1” to respondents if they attended Thanksgiving and some or no guests wore masks or socially distanced while indoors. We used this same method to create a binary indicator for risky Christmas. Lastly, we created a categorical variable for overall risky holiday behavior: 0, did not have risky attendance at either holiday; 1, attended one risky holiday; and 2, attended two risky holidays.

We examined the associations between risky holiday gathering behaviors and sociodemographic and COVID-19 risk-related variables in the UAS. Key demographic variables of interest, which we chose based on our hypothesis and the published literature, include race/ethnicity, gender**,** age, and SES of the participant (Lopez et al., 2021, Zelner et al., 2021). We used the following race/ethnicity categories: non-Hispanic white, non-Hispanic Black, Hispanic, and Other. Gender included male and female categories. We stratified age to reflect the strong age-related pattern of COVID-19 risk (i.e., <35 years, 35–44 years, 45–54 years, 55–64 years, and 65 + years). We classified SES in two ways – reported household income (<$25,000, $25,000- $49,999, $50,000- $99,999, $100,000 + ) and highest level of educational attainment (below 12th grade, high school diploma, Associate degree and some college, Bachelor’s degree, and graduate or professional degrees).

We constructed binary indicators of COVID-19 related behaviors and perceptions before the holidays from survey waves 1–16 administered between March 10, 2020 and November 11, 2020. The four behaviors (Yes = 1; No = 0) were “avoiding restaurants,” “avoided large gatherings,” “wore a mask,” and “washed hands frequently.” Additionally, we constructed a binary indicator (Yes=1; No=0) of reported COVID-19 diagnosis from a healthcare profession, which have been shown to correlate with COVID-19 related behaviors and perceptions (Gollwitzer et al., 2020). For each of the COVID-19 related behaviors and perceptions, we averaged the dichotomous scores across survey waves 1–16 for each respondent. Respondents averaging 0.5 or more were assigned ‘1’ for the given risk or perception indicator and ‘0’ otherwise.

### 2.3 Analysis

We constructed bar graphs showing the distribution of risky Thanksgiving and risky Christmas across our demographic characteristics and COVID-19 related health behaviors including washing hands frequently, avoiding high risk people, avoiding restaurants, wearing a face mask, and having a previous COVID-19 diagnosis. We tested for statistical significance with an F-test. We then replaced the household income variable with highest level of educational attainment to examine sensitivity of unadjusted SES results to an alternative measure. We assessed the robustness of unadjusted results with logistic regression models routinely used in the public health literature. In this specification, we assessed risky holiday behavior as a function of sociodemographic characteristics and several covariates including COVID-19 related behaviors. We also examined whether inference changed substantively if we combined behaviors related to Thanksgiving and Christmas into a global “risky holiday” variable (2 = risky for both holidays, 1 = risky for only one holiday, 0 = not risky for either holiday). Lastly, we conducted three sensitivity tests: 1) classifying individuals who reported most, some, or no guests wore masks and socially distanced indoors as risky (a more conservative measure of risky behavior); 2) controlling for household size as it may influence propensity to gather and socioeconomic characteristics; and 3) inserting two phases of pre-holiday behaviors as covariates to control for changes in behaviors.

UAS oversampled certain racial/ethnic and SES groups to achieve a population-representative sample. For this reason, we used UAS-assigned population weights for all analyses (but assessed sensitivity of analyses to unweighted values as well). For all analyses, we used robust standard errors to adjust for heteroscedasticity in residuals. We performed all analyses using Stata SE version 16.0. The University of California, Irvine, Institutional Review Board deemed this study exempt owing to the use of publicly available, de-identified data.

## 3 Results

**Fig. 1:**
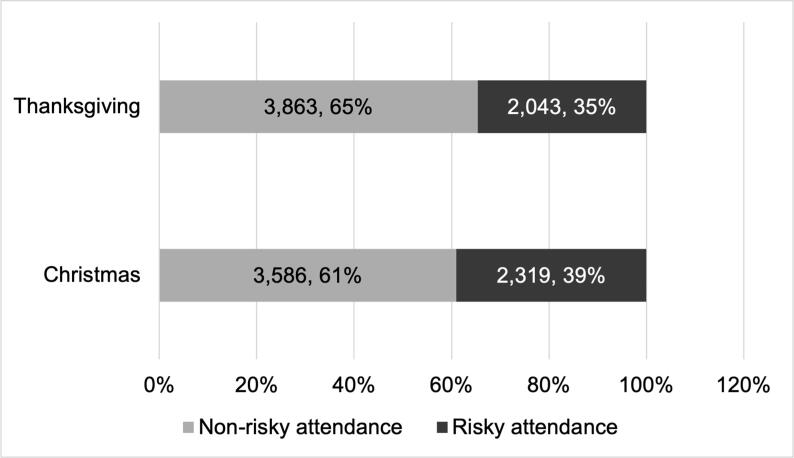
Distribution of non-risky and risky attendance at Thanksgiving and Christmas among 5,906 respondents (Thanksgiving) and 5,905 respondents (Christmas) in the Understanding America Survey, March 10, 2020 – May 25, 2021.

**Fig. 2:**
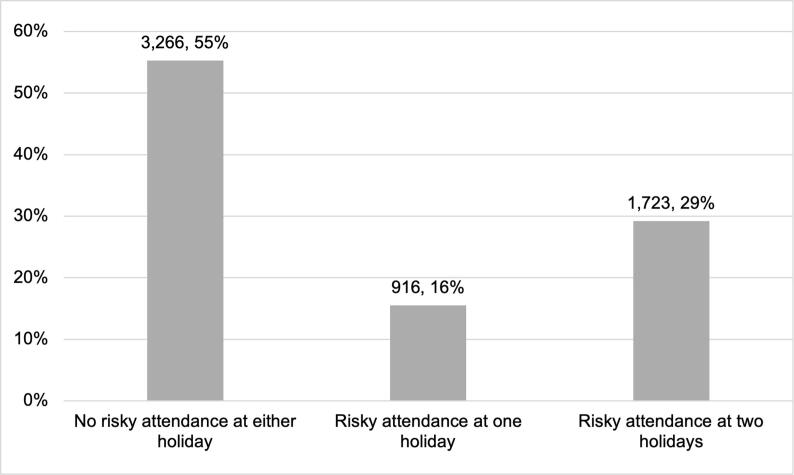
Risky attendance at neither holiday, one holiday, or two holidays among 5,905 respondents (Thanksgiving and Christmas) in the Understanding American Study, March 10, 2020 to May 25, 2021.

Fig. 1 shows risky attendance at Thanksgiving and Christmas, with 35% and 39% reporting risky behavior, respectively. In Fig. 2, the greatest proportion of participants indicate no risky behavior at either holiday (55 %), followed by those reporting risky behavior at both holidays, and participants reporting risky behavior at one holiday.

Table 1 describes sociodemographic characteristics, pre-holiday COVID-19 related behaviors, and risky holiday attendance by race/ethnicity of UAS participants. A greater proportion of non-Hispanic White individuals participate in risky behavior as opposed to other race/ethnicities (Table 1). Additionally, a greater proportion of Black, Hispanic, and Other race/ethnicities participate in protective COVID-19 related health behaviors, as compared to non-Hispanic Whites.

**Table 1:** Weighted sociodemographic and pre-holiday COVID-19 related characteristics in the total sample and by risky attendance at Thanksgiving and Christmas among 5,906 respondents (Thanksgiving) and 5,905 respondents (Christmas) in the Understanding America Study, March 10, 2020 – May 25, 2021.

Variable	All race/ethnicities	White^a^	Black^a^	Hispanic	Other^b^
	%	%	%	%	%
**Risky Thanksgiving**					
Yes	36.2	42.8	15.1	30.9	26.7
No	63.8	57.2	84.9	69.1	73.3

**Risky Christmas**					
Yes	40.7	48.2	16.0	36.6	27.4
No	59.3	51.8	84.0	63.4	72.6

**Age (years)**					
<35	23.5	20.4	22.6	31.8	30.9
35–44	22.5	20.3	24.9	28.6	23.6
45–54	15.7	14.4	19.5	16.9	17.6
55–65	17.7	18.7	18.6	15.5	14.5
65+	20.6	26.2	14.4	7.2	13.4

**Gender**					
Male	48.3	52.0	37.4	41.1	50.7
Female	51.7	48.0	62.6	58.9	49.3

**Household Income**					
<25 k	22.3	18.5	42.4	23.1	20.3
25 k-50 k	22.7	22.0	28.0	23.7	18.5
50 k-100 k	32.3	34.8	20.2	32.7	30.7
100 k+	22.7	24.7	9.4	20.5	30.5

**Washes hands**					
Yes	92.0	90.6	93.5	95.1	94.2
No	8.0	9.4	6.5	4.9	5.8

**Avoids restaurants**					
Yes	65.1	60.5	76.2	69.4	73.8
No	34.9	39.5	23.8	30.6	26.2

**Avoids risky persons**					
Yes	77.6	74.8	81.4	82.3	84.0
No	22.4	25.2	18.6	17.7	16.0

**Wears face mask**					
Yes	90.9	89.6	95.3	90.9	94.4
No	9.1	10.4	4.7	9.1	5.6

**Diagnosed with COVID-19**					
Yes	0.5	0.4	1.2	0.5	0.1
No	99.5	99.6	98.8	99.5	99.9

**Fig. 3:**
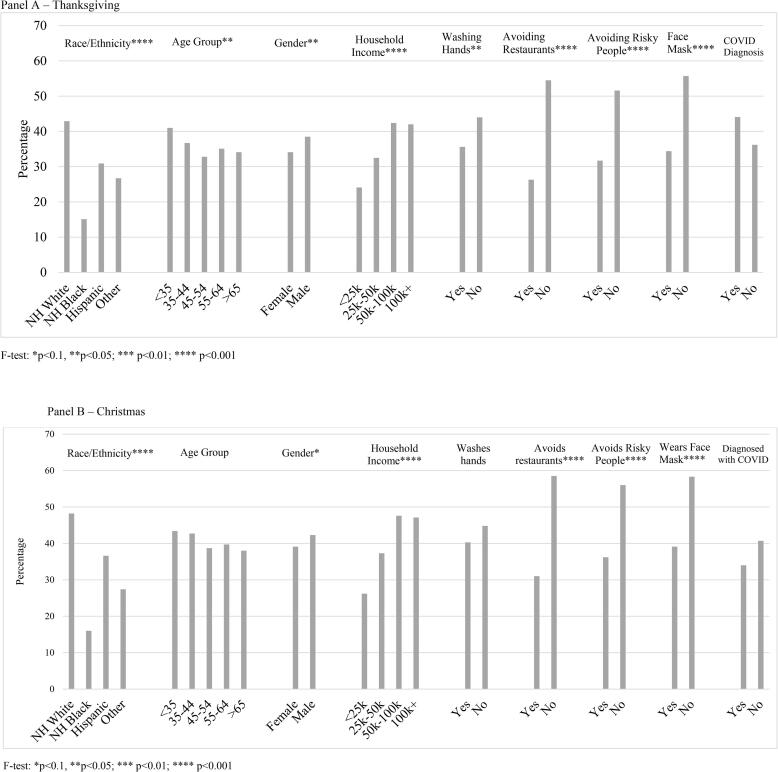
Weighted proportion of persons attending a risky Thanksgiving (Panel A) and risky Christmas (Panel B) by sociodemographic characteristics and pre-holiday COVID-19 related behaviors among 5,906 respondents (Thanksgiving) and 5,905 respondents (Christmas) in the Understanding America Study, March 10, 2020 – May 25, 2021.

Risk behaviors differed substantially by race/ethnicity (Fig. 3). For example, whereas only 15 % of NH Black respondents attended a risky Thanksgiving, 43 % of NH white respondents reported such attendance. In addition, respondents with greater household income level showed a greater prevalence of attending risky holiday gatherings. Risky attendance at Thanksgiving and Christmas was more frequent among those not participating in protective COVID-19 related behaviors (e.g., washing hands, wearing face masks). The pattern and level of statistical significance of these unadjusted results are consistent across most COVID-related behaviors reported prior to the Thanksgiving and Christmas holidays (Fig. 3).

Logistic regression analyses support the patterns found in these descriptive results (Table A2). Findings for Thanksgiving, Christmas, and the joint treatment of the holidays together showed statistically detectable, and robust, reductions in the odds of risky holiday gatherings for NH Blacks, Hispanic, and Other groups, as compared to NH Whites (Table A2, Table A3). Persons reporting household income less than $25 k (vs $100 + k) also show a lower odds of attending a risky holiday gathering. Inclusion of COVID-19 related behaviors does not substantially alter the race/ethnicity, income, gender, and age findings (Table A2). Results from the analyses using a global “risky holiday” variable for both holidays remain robust to our original findings (Table A4). Our sensitivity analyses report essentially the same results as our original findings (Appendix Table A4, Table A5, Table A6).

## 4 Conclusion

Many public health officials warned that unsafe holiday gatherings in late 2020 had the potential to produce a “winter surge” of COVID-19 infections, hospitalizations, and deaths. Whereas the COVID-19 winter surge did occur, we know of no systematic analysis of Thanksgiving and Christmas behaviors in late 2020 and whether SES and race/ethnicity—key features of the pandemic as a documented “disease of disparity”—predicted risky holiday gatherings. Results from a nationally representative survey indicate that non-Hispanic white adults, as well as those with relatively higher SES and younger age, were most likely to attend risky holiday gatherings relative to other groups. Findings indicate a highly stratified set of behaviors which support the notion that NH White and higher-income persons who may self-identify as low risk of acquiring COVID-19 appear most likely to have engaged in risky Thanksgiving and Christmas gatherings in late 2020.

The pattern of racial/ethnic results observed in our nationwide study, which controlled for a detailed set of pre-holiday behaviors related to COVID-19, appears similar to sub-national studies of holiday behavior (Peacock et al., 2020). A limitation of our study, however, involves the inability to assess whether enactment of safer holiday behaviors among racial/ethnic minorities reduced the spread of novel COVID-19 infection. Whereas the assumption that avoidance of “spreader” events such as holiday gatherings could reduce transmission, information on these dynamics of transmission were not collected. We also note the potential for variability in responses to COVID-19 related behaviors across survey waves prior to the holidays. Our use of the mean level of pre-holiday behaviors gauges the general tendency for any individual to engage in COVID-19 related protective health behaviors rather than changes in behavior over time. Additionally, data limitations do not allow us to control for baseline prevalence of holiday gatherings prior to COVID-19.

Strengths of our study include the use of a nationally representative panel of respondents spanning the entire US. Our study also leverages longitudinal aspects of the data to measure pre-holiday COVID-19 related behaviors among study respondents. Accounting for these characteristics adjusts for pre-existing tendencies toward risky behavior which may drive changes in risky holiday attendance.

Health and social inequities such as representation among essential workers or lack of access to healthcare during the pandemic may have further exacerbated disparities in COVID-19 infection (Lopez et al., 2021, CDC, 2020). One nationally representative study finds that racial/ethnic minorities, as compared to NH whites, report greater fear of coronavirus and perceive the virus as a major threat to community health (Niño et al., 2021). This perception of COVID-19 offers a plausible explanation for our results in which racial/ethnic minorities report less risky attendance at holidays. Differences in the perceived threat of COVID-19, due to disparities within these subgroups, may have altered holiday attendance and behavior.

## CRediT authorship contribution statement

**Tim A. Bruckner:** Conceptualization, Methodology, Validation, Visualization, Supervision, Project administration, Writing – original draft, Writing – review & editing, Project administration. **Abhery Das:** Methodology, Software, Formal analysis, Resources, Data curation, Writing – review & editing, Visualization. **Greg J. Duncan:** Conceptualization, Supervision, Writing – review & editing, Validation, Methodology.

## Declaration of Competing Interest

The authors declare that they have no known competing financial interests or personal relationships that could have appeared to influence the work reported in this paper.

## Figures and Tables

**Table A1:** Thanksgiving and Christmas holiday attendance and risky behaviors among 5,906 respondents in Wave 27 of the Understanding America Study, April 14, 2021 – May 25, 2021.

**Variable**	Percent of total sample
**Risk summary measures (%)**	
Risky^a^ Thanksgiving	
Yes	34.6
No	65.4
Risky^a^ Christmas	
Yes	39.3
No	60.7

**Thanksgiving risk (%)**	
Did not attend	57.5
Attended—all masked and socially distanced	4.6
Attended—some masked and socially distanced	7.6
Attended – no one masked or socially distanced	30.4

**Christmas risk (%)**	
Did not attend	59.3
Attended—all masked and socially distanced	3.7
Attended—some masked and socially distanced	6.8
Attended – no one masked or socially distanced	30.3

**Combined holiday risk (%)**	
No risky behavior^b^ at either holiday	55.3
Risky behavior^a^ at one holiday	15.5
Risky behavior^a^ at both holidays	29.2
N	5906

Over one-third of participants reported attending a risky Thanksgiving gathering and nearly 40 percent reported attending a risky Christmas gathering (Table A1). More than half of the respondents did not attend Thanksgiving or Christmas gatherings. Of those that did, very few attended either holiday with all attendees wearing masks and socially distancing. Some 29.2% of participants reported attended both a risky Thanksgiving and a risky Christmas.

a Did not attend or all attendees wore masked and socially distanced.

b Attended and some or no attendees wore masks and socially distanced.

**Table A2:** Logistic regression results predicting risky attendance at Thanksgiving or Christmas as a function of race/ethnicity (Model A), additional sociodemographic characteristics (Model B), and additional pre-holiday COVID-19 related covariates (Model C) among respondents in the Understanding America Study, March 10, 2020 – May 25, 2021.

	Thanksgiving Model A		Thanksgiving Model B		Thanksgiving Model C		Christmas Model A		Christmas Model B		Christmas Model C	
	Odds Ratio	95 % CI	Odds Ratio	95 % CI	Odds Ratio	95 % CI	Odds Ratio	95 % CI	Odds Ratio	95 % CI	Odds Ratio	95 % CI
Race/Ethnicity												
Black^b^	0.23	0.17–0.33	0.27	0.19–0.38	0.33	0.23–0.46	0.21	0.15–0.28	0.23	0.17–0.32	0.27	0.19–0.38
Hispanic^b^	0.60	0.46–0.77	0.58	0.45–0.76	0.65	0.50–0.84	0.62	0.49–0.79	0.60	0.47–0.77	0.66	0.51–0.85
Other^b^	0.48	0.36–0.65	0.44	0.33–0.60	0.50	0.37–0.68	0.40	0.30–0.54	0.37	0.28–0.51	0.41	0.30–0.56
White^b^ (ref)	–	–	–	–	–	–			–			

Gender												
Female			0.90	0.77–1.06	0.95	0.80–1.12			0.97	0.84–1.14	1.02	0.86–1.20
Male (ref)			–	–	–	–			–			

Age Group												
<35			1.61	1.27–2.04	1.24	0.96–1.60			1.48	1.17–1.87	1.16	0.91–1.50
35–44			1.25	0.99–1.58	0.97	0.76–1.24			1.36	1.08–1.71	1.08	0.85–1.38
45–54			1.08	0.84–1.38	0.87	0.68–1.13			1.19	0.93–1.51	0.98	0.76–1.26
55–64			1.14	0.91–1.43	1.06	0.84–1.34			1.16	0.93–1.45	1.08	0.86–1.36
65+ (ref)			–	–	–	–						

Household Income												
<25 k			0.52	0.85–1.26	0.46	0.35–0.60			0.47	0.37–0.60	0.43	0.33–0.56
25 k-50 k			0.73	0.58–0.92	0.69	0.54–0.88			0.74	0.59–0.93	0.70	0.56–0.89
50 k-100 k			1.03	0.40–0.66	0.98	0.80–1.20			1.04	0.86–1.27	0.99	0.81–1.21
100 k+ (ref)			–	–	–	–						

Fox news as a source for COVID-19												
Yes					1.03	0.83–1.26					1.02	0.83–1.25
No (ref)					–	–						

Washing hands frequently												
Yes					1.53	1.07–2.20					1.78	1.25–2.54
No (ref)					–	–						

Avoiding restaurants												
Yes					0.36	0.30–0.44					0.39	0.32–0.47
No (ref)					–	–						

Avoiding contact with high-risk people												
Yes					0.75	0.60–0.94					0.70	0.51–1.02
No (ref)					–	–						

Wearing a face mask												
Yes					0.69	0.49–0.98					0.71	0.56–0.89
No (ref)					–	–						

Diagnosed with COVID-19 by a healthcare professional												
Yes					2.39	0.60–9.56					1.17	0.29–4.70
No (ref)												
N	5,897		5,897		5,897		5,896		5,896		5,896	

**Table A3:** Ordered logistic regression results predicting combined risky attendance at Thanksgiving and Christmas holidays as a function of race/ethnicity (Model A), additional sociodemographic characteristics (Model B), and additional pre-holiday COVID-19 related covariates (Model C) among respondents in the Understanding America Study, March 10, 2021 – May 25, 2021.

	Model A		Model B		Model C	
	Odds Ratio	95 % CI	Odds Ratio	95 % CI	Odds Ratio	95 % CI
Race/Ethnicity						
Black^b^	0.22	0.17–0.30	0.25	0.19–0.34	0.30	0.21–0.40
Hispanic^b^	0.59	0.46–0.75	0.57	0.44–0.73	0.63	0.50–0.81
Other^b^	0.44	0.34–0.57	0.40	0.30–0.52	0.44	0.33–0.58
White^b^ (ref)	–	–	–	–	–	–

Gender						
Female			0.94	0.81–1.09	0.99	0.85–1.16
Male (ref)			–	–	–	–

Age Group						
<35			1.57	1.25–1.96	1.22	0.96–1.54
35–44			1.32	1.06–1.65	1.03	0.82–1.30
45–54			1.15	0.91–1.45	0.94	0.74–1.18
55–64			1.13	0.92–1.40	1.06	0.85–1.31
65+ (ref)			–	–	–	–

Household Income						
<25 k			0.48	0.38–0.61	0.43	0.34–0.55
25 k-50 k			0.74	0.60–0.92	0.70	0.56–0.88
50 k-100 k			1.05	0.87–1.25	0.99	0.82–1.19
100 k+ (ref)			–	–	–	–

Fox news as a source for COVID-19						
Yes					1.01	0.83–1.21
No (ref)					–	–

Washing hands frequently						
Yes					1.66	1.19–2.31
No (ref)					–	–

Avoiding restaurants						
Yes					0.37	0.31–0.44
No (ref)					–	–

Avoiding contact with high-risk people						
Yes					0.72	0.59–0.88
No (ref)					–	–

Wearing a face mask						
Yes					0.68	0.48–0.95
No (ref)					–	–

Diagnosed with COVID-19 by a healthcare professional						
Yes					1.79	0.48–6.68
No (ref)					–	–
N	5,896	5,896	5,896			

**Table A4:** Logistic regression results predicting risky attendance at Thanksgiving or Christmas, classifying individuals who indicated that most, some, or no guests wore masks and socially distanced as risky, as a function of race/ethnicity, sociodemographic characteristics, and pre-holiday COVID-19 related covariates among respondents in the Understanding America Study, March 10, 2020 – May 25, 2021.

	Thanksgiving		Christmas	
	Odds Ratio	95 % CI	Odds Ratio	95 % CI
Race/Ethnicity				
Black^b^	0.37	0.26–0.51	0.30	0.22–0.41
Hispanic^b^	0.67	0.52–0.87	0.68	0.53–0.88
Other^b^	0.57	0.42–0.77	0.44	0.33–0.60
White^b^ (ref)	–	–	–	–

Gender				
Female	0.96	0.81–1.13	1.08	0.92–1.28
Male (ref)	–	–	–	–

Age Group				
<35	1.29	0.80–1.27	1.24	0.98–1.60
35–44	1.05	0.78–1.30	1.10	0.87–1.41
45–54	1.01	0.82–1.34	1.07	0.83–1.36
55–64	1.01	0.98–1.66	1.06	0.85–1.33
65+ (ref)	–	–	–	–

Household Income				
<25 k	0.52	0.40–0.67	0.46	0.36–0.60
25 k-50 k	0.75	0.59–0.95	0.71	0.56–0.90
50 k-100 k	1.04	0.85–1.27	0.96	0.81–1.21
100 k+ (ref)	–	–	–	–

Washing hands frequently				
Yes	1.40	0.98–1.99	1.60	1.12–2.28
No (ref)	–	–	–	–

Avoiding restaurants				
Yes	0.37	0.30–0.44	0.39	0.32–0.47
No (ref)	–	–	–	–

Avoiding contact with high-risk people				
Yes	0.74	0.59–0.92	0.72	0.57–0.91
No (ref)	–	–	–	–

Wearing a face mask				
Yes	0.69	0.49–0.98	0.66	0.56–0.95
No (ref)	–	–	–	–

Diagnosed with COVID-19 by a healthcare professional				
Yes	2.39	0.60–9.56	1.06	0.27–4.04
No (ref)	–	–	–	–
N	5,765		5,781	

**Table A5:** Logistic regression results predicting risky attendance at Thanksgiving or Christmas, controlling for household size, as a function of race/ethnicity, sociodemographic characteristics, and additional pre-holiday COVID-19 related covariates among respondents in the Understanding America Study, March 10, 2020 – May 25, 2021.

	Thanksgiving		Christmas	
	Odds Ratio	95 % CI	Odds Ratio	95 % CI
Race/Ethnicity				
Black^b^	0.33	0.23–0.48	0.28	0.19–0.39
Hispanic^b^	0.66	0.50–0.86	0.66	0.51–0.86
Other^b^	0.52	0.38–0.71	0.43	0.31–0.58
White^b^ (ref)	–	–	–	–

Gender				
Female	0.96	0.81–1.14	1.02	0.87–1.21
Male (ref)	–	–	–	–

Age Group				
<35	1.26	0.96–1.65	1.15	0.84–1.33
35–44	0.99	0.76–1.29	1.07	0.76–1.28
45–54	0.89	0.68–1.15	0.99	0.82–1.39
55–64	1.04	0.82–1.32	1.05	0.88–1.49
65+ (ref)	–	–	–	–

Household Income				
<25 k	0.47	0.36–0.62	0.46	0.35–0.60
25 k-50 k	0.69	0.54–0.88	0.73	0.57–0.93
50 k-100 k	0.98	0.80–1.20	1.01	0.82–1.22
100 k+ (ref)	–	–	–	–

Washing hands frequently				
Yes	1.52	1.06–2.17	1.77	1.24–2.53
No (ref)	–	–	–	–

Avoiding restaurants				
Yes	0.36	0.30–0.44	0.39	0.32–0.47
No (ref)	–	–	–	–

Avoiding contact with high-risk people				
Yes	0.74	0.59–0.93	0.69	0.55–0.86
No (ref)	–	–	–	–

Wearing a face mask				
Yes	0.69	0.49–0.97	0.72	0.51–1.03
No (ref)	–	–	–	–

Diagnosed with COVID-19 by a healthcare professional				
Yes	2.60	0.60–11.23	1.22	0.29–5.17
No (ref)	–	–	–	–
Number of Household members	0.99	0.94–1.07	1.02	0.95–1.08
N	5,745		5,744	

**Table A6:** Logistic regression results predicting risky attendance at Thanksgiving or Christmas, controlling Phase 1 (Waves 1–8) and Phase 2 (Waves 9–16) pre-holiday behaviors, as a function of race/ethnicity, and sociodemographic characteristics among respondents in the Understanding America Study, March 10, 2020 – May 25, 2021.

	Thanksgiving		Christmas	
	Odds Ratio	95 % CI	Odds Ratio	95 % CI
Race/Ethnicity				
Black^b^	0.35	0.23–0.50	0.29	0.20–0.42
Hispanic^b^	0.71	0.54–0.94	0.69	0.53–0.89
Other^b^	0.55	0.39–0.76	0.43	0.31–0.60
White^b^ (ref)	–	–	–	–

Gender				
Female	0.99	0.83–1.18	1.03	0.86–1.22
Male (ref)	–	–	–	–

Age Group				
<35	1.17	0.89–1.54	1.13	0.86–1.47
35–44	0.89	0.69–1.15	1.01	0.79–1.30
45–54	0.82	0.63–1.06	0.92	0.71–1.19
55–64	1.05	0.82–1.33	1.08	0.85–1.37
65+ (ref)	–	–	–	–

Household Income				
<25 k	0.40	0.30–0.52	0.37	0.28–0.48
25 k-50 k	0.61	0.47–0.78	0.64	0.50–0.82
50 k-100 k	0.92	0.74–1.13	0.96	0.78–1.18
100 k+ (ref)	–	–	–	–

Washing hands frequently (Waves 1–8)				
Yes	0.93	0.50–1.73	0.99	0.54–1.82
No (ref)	–	–	–	–

Washing hands frequently (Waves 9–16)				
Yes	1.30	0.80–2.13	1.58	0.98–2.56
No (ref)	–	–	–	–

Avoiding restaurants (Wave 1–8)				
Yes	0.81	0.63–1.06	0.90	0.69–1.16
No (ref)	–	–	–	–

Avoiding restaurants (Wave 9–16)				
Yes	0.37	0.30–0.45	0.35	0.28–0.43
No (ref)	–	–	–	–

Avoiding contact with high-risk people (Waves 1–8)				
Yes	1.18	0.85–1.63	1.01	0.73–1.40
No (ref)	–	–	–	–

Avoiding contact with high-risk people (Waves 9–16)				
Yes	0.89	0.67–1.19	0.80	0.60–1.06
No (ref)	–	–	–	–

Wearing a face mask (Waves 1–8)				
Yes	0.59	0.45–0.76	0.71	0.55–0.92
No (ref)	–	–	–	–

Wearing a face mask (Waves 9–16)				
Yes	1.01	0.70–1.46	1.04	0.71–1.51
No (ref)	–	–	–	–

Diagnosed with COVID-19 by a healthcare professional (Waves 1–8)				
Yes	1.56	1.03–2.35	1.52	1.02–2.27
No (ref)	–	–	–	–

Diagnosed with COVID-19 by a healthcare professional (Waves 9–16)				
Yes	1.66	0.78–3.54	1.74	0.88–3.45
No (ref)	–	–	–	–
N	5,495		5,494	

## Appendix

### Background on the Understanding America Study (UAS)

As a probability-based online panel, the Understanding America Study (UAS) began in 2014 and is administered by the Center for Economic and Social Research at the University of Southern California (USC). It remains representative of the adult US population with respect to sociodemographic characteristics. UAS has more than 9,000 panel members with all surveys available in English and Spanish. The UAS recruited households through a multi-phase, address-based sampling frame through mail and phone-based methods. The study randomly selects addresses from zip codes across the US and adjusts each wave to account for non-response in the previous wave. If certain zip codes have a greater proportion of non-response, they are over-sampled to match population proportions. UAS recruits participants by mailing informational packets and follows up with non-responders by mail and phone. Responders receive a refillable debit card at that time. For the nationally representative survey, UAS recruited approximately 10,329 individuals and 18.9 % became panel members. Completion for each survey wave ranges from 70 to 95 % and respondents are compensated $20 for each 30-minute survey. For retainment, UAS continues to contact panel members for 10 months following non-response. Six weeks after the 10-month period, respondents receive a termination letter indicating that they can continue UAS membership if they wish to return.

The survey provided internet-connected tablets as needed so panel membership did not remain dependent on a survey respondent’s Internet access. Of the active panel members, approximately 5.2 % received tablets connected to the Internet (compared to ∼ 15 % of the US population that does not have internet). UAS recruited individuals without internet at half the rate of individuals with internet.

For weighting, the survey first creates base weights, to adjust for differences in sampling. The UAS computes base weights by zip code and estimates the probability of sampling as a function of urbanicity, population size, sex, race/ethnicity, age, marital status, and education composition. The study utilizes the American Community Survey (ACS) to estimate base weights. Second, the survey creates final post-stratification weights – allowing the study sample for each survey to remain nationally representative. UAS performs raking weighting (marginal weighting) to assign post-stratification weights. This aligns the final survey to the reference population using the US Census Basic Monthly Current Population Survey (CPS). UAS utilizes the six most recent monthly CPS surveys to ensure consistency in respondents and benchmark populations. The study utilizes weights for all UAS panel members except for respondents recruited through American Indian samples who do not identify as American Indian (mistakenly sampled) and women who have given birth in LA county between 2009 and 2012 (sampled for a specific nutrition survey).

Reference:

Understanding America Study. Accessed May 13, 2022. https://uasdata.usc.edu/index.php.

## References

[b0005] Carroll R., Prentice C.R. (2021). Community vulnerability and mobility: What matters most in spatio-temporal modeling of the COVID-19 pandemic?. Soc. Sci. Med..

[b0010] CDC. Health equity considerations and racial and ethnic minority groups. Centers for Disease Control and Prevention. Published February 11, 2020. Accessed November 29, 2021. https://www.cdc.gov/coronavirus/2019-ncov/community/health-equity/race-ethnicity.html.

[b0015] Cohen P.N., Casper L.M. (2002). In Whose Home? Multigenerational Families in the United States, 1998–2000. Sociol. Perspect..

[b0020] Cross C.J. (2018). Extended family households among children in the United States: Differences by race/ethnicity and socio-economic status. Popul. Stud..

[b0025] CDC. COVID Data Tracker. Centers for Disease Control and Prevention. Published March 28, 2020. Accessed October 26, 202 https://covid.cdc.gov/covid-data-tracker.

[b0030] Fernandez, M., Holder, J., Leatherby, L., Valentino-DeVries, J., Tompkins, L., 2020. As Christmas Nears, Virus Experts Look for Lessons From Thanksgiving. *The New York Times*. https://www.nytimes.com/interactive/2020/12/20/us/covid-thanksgiving-effect.html. Published December 20, 2020. Accessed November 29, 2021.

[b0035] Gollwitzer A., Martel C., Brady W.J. (2020). Partisan differences in physical distancing are linked to health outcomes during the COVID-19 pandemic. Nat. Hum. Behav..

[b0040] Groves, S. Data shows Americans couldn’t resist Thanksgiving travel. AP NEWS. Published April 20, 2021. Accessed October 26, 2021. https://apnews.com/article/data-americans-thanksgiving-travel-209e88a889664ad5e25a81ee0d46201c.

[b0045] Lopez L., Hart L.H., Katz M.H. (2021). Racial and Ethnic Health Disparities Related to COVID-19. JAMA..

[b0050] Niño M., Harris C., Drawve G., Fitzpatrick K.M. (2021). Race and ethnicity, gender, and age on perceived threats and fear of COVID-19: Evidence from two national data sources. SSM – Popul. Health..

[b0055] Peacock, J.E., Herrington, D.M., Edelstein, S.L., et al., 2020. Survey of Adherence with COVID-19 Prevention Behaviors During the 2020 Thanksgiving and Winter Holidays Among Members of the COVID-19 Community Research Partnership. J Community Health. Published online August 12, 2021. doi:10.1007/s10900-021-01021-z. 10.1007/s10900-021-01021-z PMC8358902 34383157

[b0060] Rosenstock I.M. (1974). The health belief model and preventive health behavior. Health Educ. Monogr..

[b0065] Taylor R.J., Chatters L.M., Woodward A.T., Brown E. (2013). Racial and ethnic differences in extended family, friendship, fictive kin and congregational informal support networks. Fam. Relat..

[b0070] Transportation Security Administration. TSA checkpoint travel numbers (current year versus prior year(s)/same weekday) | Transportation Security Administration. Published 2021. Accessed October 26, 2021. https://www.tsa.gov/coronavirus/passenger-throughput.

[b0075] Understanding America Study. Published 2021. Accessed December 1, 2020. https://uasdata.usc.edu/index.php.

[b0080] Zelner J., Trangucci R., Naraharisetti R. (2021). Racial Disparities in Coronavirus Disease 2019 (COVID-19) Mortality Are Driven by Unequal Infection Risks. Clin. Infectious Dis..

